# Individualizing treatment targets for elderly patients with type 2 diabetes: factors influencing clinical decision making in the 24-week, randomized INTERVAL study

**DOI:** 10.18632/aging.101188

**Published:** 2017-03-05

**Authors:** W. David Strain, Abhijit S. Agarwal, Päivi M. Paldánius

**Affiliations:** ^1^ Diabetes and Vascular Research Centre, University of Exeter Medical School, Exeter EX2 5AX, UK; ^2^ Novartis Pharmaceuticals Corporation, East Hanover, NJ 07936-1080, USA; ^3^ Novartis Pharma AG, Postfach, CH-4002 Basel, Switzerland

**Keywords:** elderly, individualization, predictors, type 2 diabetes

## Abstract

We tested the feasibility of setting individualized glycemic goals and factors influencing targets set in a clinical trial in elderly patients with type 2 diabetes.

A 24-week, randomized, double-blind, placebo-controlled study was conducted in 45 outpatient centers in seven European countries. 278 drug-naïve or inadequately controlled (mean HbA1c 7.9%) patients with type 2 diabetes aged ≥70 years with HbA1c levels ≥7.0% and ≤10.0% were enrolled. Investigator-defined individualized HbA1c targets and the impact of baseline characteristics on individualized treatment targets was evaluated.

The average individualized HbA1c target was set at 7.0%. HbA1c at baseline predicted a target setting such that higher the HbA1c, more aggressive was the target (P<0.001). Men were more likely to be set aggressive targets than women (P=0.026). Frailty status of patients showed a trend towards significance (P=0.068), whereas diabetes duration, age, or polypharmacy did not. There was heterogeneity between countries regarding how baseline factors were viewed.

Despite training and guidance to individualize HbA1c goals, targets were still set in line with conventional values. A strong influence of country-specific guidelines on target setting was observed; confirming the importance of further education to implement new international guidelines in older adults.

## INTRODUCTION

Type 2 diabetes is one of the most common chronic diseases in older populations, affecting ~ 20.0% of individuals with age >75 years [1, 2]. A considerable proportion of these older individuals have multiple comorbidities due in part to their longevity [3, 4]. Older individuals with diabetes have significantly increased risk of microvascular and macrovascular disease, cognitive dysfunction, functional impairment, depression, and vision and hearing impairment compared with younger adults [5, 6]. Further, the high prevalence of polypharmacy in elderly patients exposes them to a greater risk of complications and adverse reactions to any new pharmaceutical intervention [6]. However, frailty and multiple comorbidities have led to the exclusion of elderly patients from a majority of clinical trials of glycemic therapeutics until recently [6–9]. The recent global guidelines for the treatment of elderly patients have emphasized on the need of a holistic and individualized approach to patient management and setting appropriate targets for this population [2, 6–10]. However, there is no evidence to date that setting these individualized targets is even feasible, let alone assessing whether they can be achieved or improve outcomes [5, 6, 9, 10].

The INdividualized Treatment targets for EldeRly patients with type 2 diabetes using Vildagliptin Add-on or Lone therapy (INTERVAL) study was the first, and to date the only, clinical study that pragmatically assessed the feasibility of setting and achieving investigator-defined individualized treatment targets in elderly patients with type 2 diabetes [11]. Despite the guidance to set individualized targets based on patients' comorbidities and baseline characteristics and the training provided to facilitate this endeavor, the mean individualized HbA1c target set was 7.0%, identical to the contemporaneous conventional guidelines.

Current guidelines advocate individualizing goals, yet our investigators, with a particular interest in diabetes in older adults and despite specific training in establishing these targets, deviated only marginally from conventional targets. To understand the factors that may hinder the application of global guidelines to individualize goals, we now review the targets set by these trained investigators, the determinants of those targets and the factors impacting HbA1c reduction.

## RESULTS

The study enrolled 278 patients in total. Patients' demographic characteristics have been presented in detail elsewhere [11]. In brief, 152 (54.7%) patients were female, 124 (44.6%) patients were aged ≥75 years and 26 (9.4%) patients reached the stringent criteria for frail (although physicians regarded more of their patients as frail according to general clinical judgement). The mean (standard deviation) age was 74.8 (4.17) years (range, 70–97 years) and body mass index 29.8 (4.34) kg/m^2^. The mean (standard deviation; range) HbA1c was 7.9% (0.72; 6.6% to 10.3%), with 173 (62.2%) patients with HbA1c levels of ≤8.0%, despite a mean (standard deviation; range) duration of diabetes of 11.4 years (7.47; 0.3 to 35.0 years). The patients were taking an average of six (range, 1–15) different medications, with a substantially higher tablet burden, before randomization to study drug or placebo.

**Figure 1:**
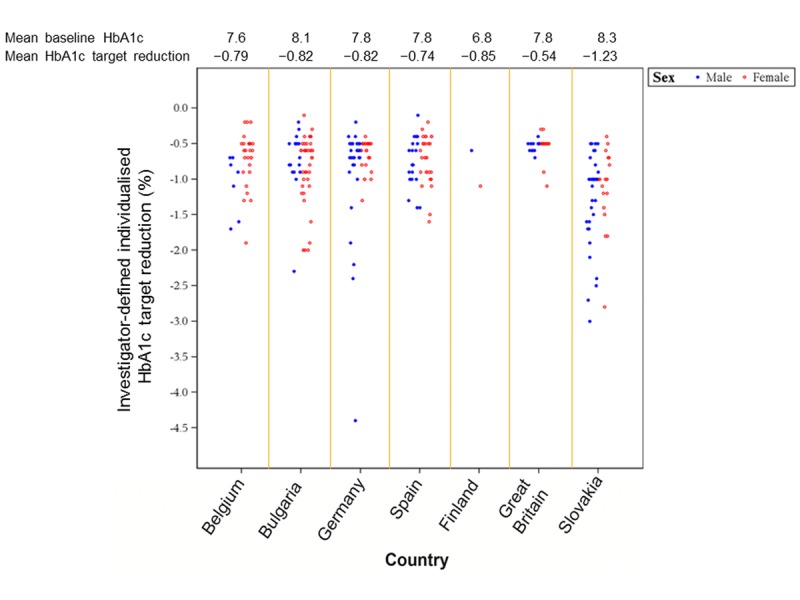
Summary of individualized HbA1c targets set by investigators (by country)

A summary of the individualized HbA1c targets set by the investigators by countries is provided in Figure 1. The mean overall HbA1c target reduction was −0.9% (range, −4.4% to −0.1%). In patients with HbA1c up to 8.0%, the mean individualized target reduction was less stringent, −0.7% (range, −2.4% to −0.1%), whereas in patients with HbA1c >8.0% the mean individual target reduction was −1.2% (range, −4.4% to −0.2%).

**Figure 2:**
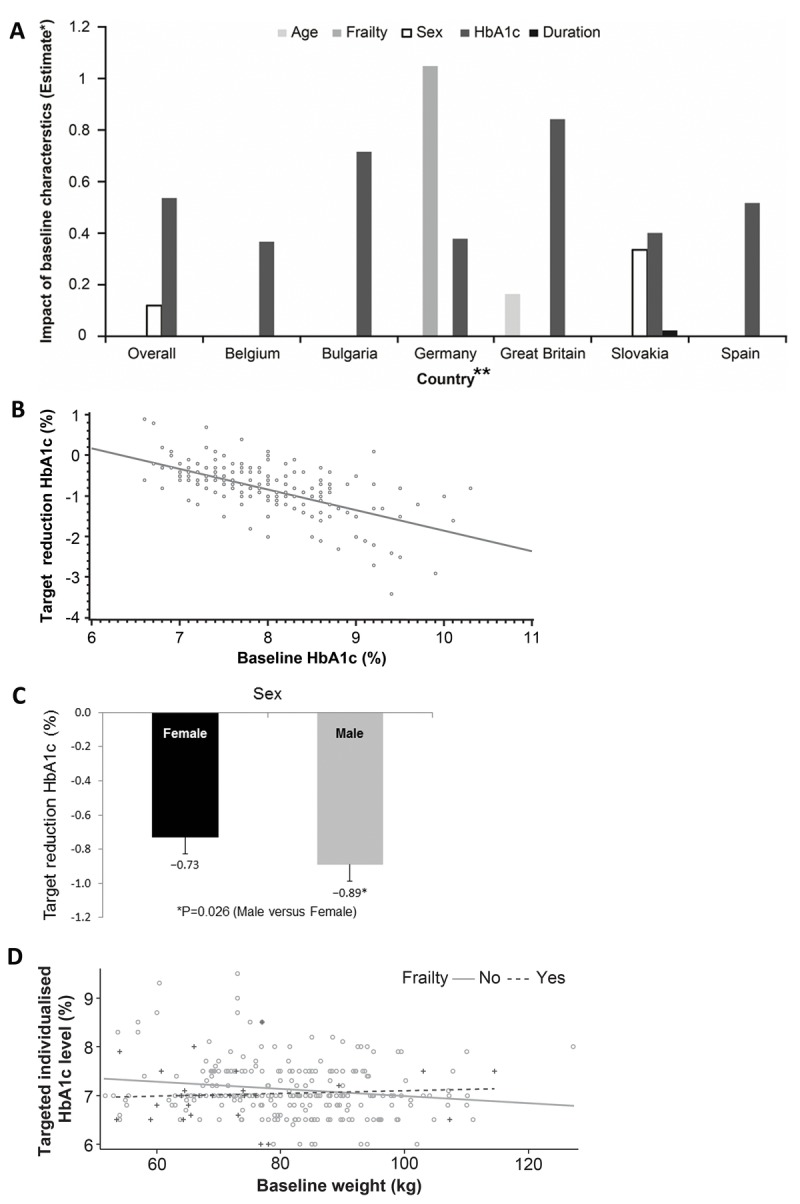
**(A)** Baseline factors affecting target setting (overall and by country). *For categorical covariates, the estimate is the difference between the adjusted means of comparison-reference in the corresponding category. For continuous covariates, the estimate is the change in adjusted means per unit. **Patients from Finland were identified by a single investigator. The figure estimates the difference between adjusted means for different factors potentially driving the individualized target setting and thus no reliable statistics for such a low sample size (n=2) could be generated. Hence, Finland has been removed. **(B)** Baseline HbA1c versus target reduction HbA1c. **(C)** Sex status versus target reduction HbA1c. **(D)** Baseline weight versus targeted individualized HbA1c by frailty status.

The impact of baseline characteristics on target setting, overall and by country, is summarized in Figure 2a. In the overall study, screening HbA1c was positively associated with the target reduction such that for every 1% increase in the baseline HbA1c, the target reduction was increased by −0.5% (Pearson's correlation −0.6353; P<0.001; Figure 2b). Men were set more aggressive targets than women (P=0.026; Figure 2c), whereas the frailty status only demonstrated a trend towards significance (P=0.068). In non-frail patients, the baseline weight was a predictor of a less aggressive glycemic target setting (P=0.012) such that more obese patients were set more aggressive targets, while in frail patients, a lower body weight did not additionally impact the glycemic target (P=0.725; Figure 2d). Interestingly and unexpectedly, neither age (P=0.510) nor the duration of diabetes (P=0.760) had an impact on the targets set. Hematological and biochemical parameters also did not seem to predict the target established. Physicians did not seem to consider the degree of polypharmacy when setting targets (P=0.301); the addition of the number of concomitant prescriptions and other medications to the analysis model did not significantly alter any of the associations.

When exploring determinants at the country level, screening HbA1c was the only universal factor affecting target setting (P<0.001). Sex was a significant factor in Slovakia (P=0.025) and showed a trend in Germany (P=0.057), similar to the overall study. The frailty status was a significant factor only in Germany (P=0.002), while it also showed a trend in Belgium (P=0.085). Age was a significant factor only in Great Britain (P=0.025), whereas duration of diabetes was a significant factor only in Slovakia (P=0.018).

**Figure 3:**
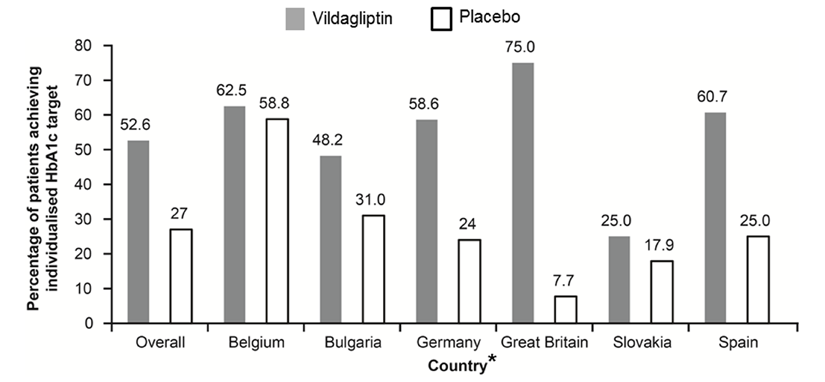
Summary of individualized HbA1c target response (overall and by country) *Patients from Finland were identified by a single investigator. The figure estimates the difference between adjusted means for different factors potentially driving the individualized target setting and thus no reliable statistics for such a low sample size (n=2) could be generated. Hence, Finland has been removed.

As previously reported, the adjusted odds ratio of achieving the individualized target in the overall study population was 3.16 (P<0.001) with study drug, vildagliptin, compared to placebo [11]. This was on a background of 37 (27%) participants achieving their target on placebo alone. Great Britain had the highest odds ratio (59.22; 95% confidence interval 3.00 to 1168.96; P=0.007) driven predominantly by the low percentage of patients in the placebo group achieving their individualized targets (7.7%). Belgium, on the other hand, with the highest percentage of patients achieving targets with placebo alone (58.8%), had the least relative benefit by introduction of medication (odds ratio 1.13; 95% confidence interval 0.28 to 4.63; P=0.862) (Figure 3).

## DISCUSSION

Although global guidelines for the management of type 2 diabetes advocate individualization of target setting, to date, the INTERVAL study is the first and only clinical study to explore the feasibility of setting such targets, let alone the evaluation of achieving these in a clinical setting. Despite extensive training and guidance to regard local guidelines as a secondary consideration, in our study, physicians maintained the traditional goal of 7.0% for this elderly and frail cohort. Further, this study is the first to explore the independent determinants of the targets that were set and, thereby, determine the specific areas of education that may be required to facilitate more appropriate target setting for elderly adults with type 2 diabetes. Guidelines published by the European Diabetes Working Party for Older People (aged ≥70 years) [9], the Consensus Development Conference on Diabetes and Older Adults (aged ≥65 years) convened by the American Diabetes Association [6], the Position Statement of the American Diabetes Association and the European Association for the Study of Diabetes [3] and, more recently, the International Diabetes Federation [2], all recommend an HbA1c target of 7.0% to 7.5% for elderly patients with type 2 diabetes without major comorbidities and 7.6% to 8.5% for frail, dependent patients with multiple comorbidities and high risk of hypoglycemia. These recommendations are based on opinions of the respective committees and experts rather than actual clinical evidence [12], indeed, the limited data used for formulating these recommendations were extrapolated from younger patients as most clinical trials exclude frail elderly patients with polypharmacy and multiple comorbidities [2, 6, 9]. Furthermore, these guidelines also suggest a patient-centered approach by providing the patient and caregiver structured education about the disease and treatment options and taking into account their individual medical, social, and cultural circumstances. Each patient should have the opportunity to make an informed decision regarding their treatment targets and therapeutic options including lifestyle modifications and pharmaceutical interventions [7, 8]. This is similar to the guidance and training provided to the investigators in the INTERVAL study, all of whom had special interest in diabetes in the elderly. Therefore, the conventional target of 7.0% that was set here was cause for concern and suggests that significant additional resources will be required to facilitate a change in clinical practice in a wider arena. We suggest that the introduction of guidelines alone may be insufficient to change attitudes and establish individualized care that these guidelines are hoping to achieve.

The physicians in one or more centers in some countries seemed to set rigid, particularly aggressive and uniform HbA1c targets even in these elderly patients with type 2 diabetes. The median and mean target reductions in these countries were similar for all patients suggesting that these aggressive targets were not a response to differing baseline characteristics such as comorbidities and duration of disease but a blanket adherence to more historic, aggressive HbA1c targets.

Unexpectedly, there was a paradoxical association between sex of the participant and individualized target set, with more aggressive targets being set for men compared with women, despite a higher attributable risk of hyperglycemia to the adverse consequences of diabetes in women than men [13–16]. One likely explanation for this finding may be that elderly women with diabetes were potentially perceived to be more prone to falls and fractures [3, 17], and investigators potentially wished to avoid hypoglycemia by setting less stringent targets. Alternatively, this finding could also be attributed to the incorrect assumption that older men have a higher risk of cardiovascular disease and, therefore, require more aggressive treatment [18, 19]. Either way significant education is required here.

The frailty status showed a tendency towards significance in the overall population, but that may be attributed in part to stringency of the modified Fried criteria that were used to define frailty in this study. The lack of association between age or duration of diabetes and targets set may represent the distinction that occurs in older adults compared with younger populations, where chronological age is discounted in preference of biological age. We had no way of capturing this subjective and, often, subconscious assessment by our investigators. However, given the overall aggressive targets that were set, this could represent the direct converse; physicians today disregard age when setting targets for fear of being accused of discriminating against people based on age [20]. This is a significant barrier to optimizing treatment for elderly adults.

Multiple co-morbidities and polypharmacy are other important factors in the management of older adults. Drug-drug interactions, increased risk of side effects particularly in patients with renal impairment, decreased adherence, less clinical benefit from otherwise appropriate medicines, and an increased risk of falling in older patients are all important considerations [6]. The study population was representative of the real world elderly patients with type 2 diabetes with the majority having multiple comorbidities and an average of six and up to 15 co-prescriptions pre-randomization, many with multiple daily dosing, further translating into a higher volume of tablets per person. Unpredictably, the number of medications did not impact individualized target setting in this study. This may be due to the perceived safety profile of the active agent, vildagliptin; however, it was disappointing that the implied multiple co-morbidities that accompany polypharmacy were not a consideration.

Nevertheless, choosing an optimal target is not always easy. Lack of consensus even among internationally acknowledged diabetes experts in weighing appropriateness of factors for setting glycemic targets for individual patients has led to exploration of usefulness of a survey-based algorithm for target setting [21]. The proposed algorithm suggests considering factors, such as life expectancy and risk of hypoglycemia, as main drivers of target setting while resource setting or disease duration *per se* are considered less important [21]. However, despite helpful tools, such as algorithms, assessment of cognitive function or patient's adherence to therapy remain subjective, confirming that individualized target setting is still an art.

Findings from the Diabetes Prevention Program suggested that adults aged ≥60 years showed better efficacy from lifestyle interventions than younger patients with type 2 diabetes [22]. This could explain the results from the INTERVAL study in which all patients seemed to benefit from the opportunity to interact with their physicians while setting their individualized treatment targets as evidenced in particular by the high percentage of patients (27%) in the placebo arm who reached their individualized targets. However, the proportion of patients in the placebo group achieving their individualized targets varied remarkably across countries. This may be due to variation in the background education provided to older adults. Adequate advice regarding lifestyle, diet and exercise prior to their enrolment into the study would tend to attenuate the placebo effect. In other centers, where the perception may be that elderly adults may not benefit from such advice or a system does not support such education, enrolment into the study would tend to generate an exaggerated efficacy of the patient engagement. We do acknowledge the small sample size of patients from each country limits the application of these results as representative of the entire country; however, they do provide a unique glimpse of the challenges in the synchronization of global guidelines with local clinical practices.

The INTERVAL study introduced a unique endpoint of individualized glycemic treatment targets to guide the “real-life” approach for treatment of elderly patients with type 2 diabetes, and reflected the unmet clinical need to understand the importance of individualized target setting, particularly in a more fragile population. No studies on individualized treatment targets or assessments of tolerability of any individualized treatments have been reported prior to this study. The INTERVAL study was exploratory, and further work will be required to better understand the consequences of such individualization of glycemic targets and the determinants of the glycemic targets set.

In conclusion, INTERVAL was the first study to explore the feasibility of setting individualized targets when managing diabetes in the growing elderly population. In our population of trained and motivated investigators, individualized treatment targets were disappointingly aligned with conventional guideline targets. Therefore, we suggest significant investment in the implementation and adaptation of the ubiquitous global treatment guidelines to personalize medicine in any population will be required before we can truly offer individualized care.

## MATERIALS AND METHODS

### Study design and patient population

This was a 24-week, randomized, double-blind, placebo-controlled study conducted at 45 outpatient centers in seven European countries (Belgium, Bulgaria, Germany, Finland, Slovakia, Spain, and United Kingdom) between 22 December 2010 and 14 March 2012. Drug-naïve or inadequately controlled patients with type 2 diabetes aged ≥70 years with HbA1c levels ≥7.0% and ≤10.0% at the screening visit were eligible to participate in this study. The frailty status of patients was evaluated using a modified version of the criteria proposed by Fried and colleagues [23]. Patients were considered frail if they met any two of the following three criteria: unintentional weight loss, slow walking speed, and poor grip strength as measured by a dynamometer.

Study investigators were trained in individualizing treatment targets based on their clinical judgement considering characteristics, such as age, frailty, comorbidities, and baseline HbA1c values. During the randomization visit, each patient agreed to an individualized 24-week HbA1c target with the investigator. Patients were provided with information about the meaning of their individualized treatment targets, triggers and symptoms of hypoglycemia, and appropriate treatment for adverse events. However, no formal diabetes education program was engaged, and agreement was sought from participants to maintain their current diet and exercise habits for the duration of the study. Patients were randomized in a 1:1 ratio to receive either vildagliptin (according to the label) or placebo. Study analyses were performed on the full analysis set (FAS), comprising all randomized patients. Further details of the study population and design are described elsewhere [11].

### Study assessments and endpoints

We have previously reported the co-primary endpoints of proportion of patients reaching their investigator-defined HbA1c target and reduction in the HbA1c value from baseline to week 24. The a priori secondary analyses presented herein evaluated the individualized HbA1c targets set by the investigators and the impact of baseline characteristics on these targets. Further, the response from baseline of the co-primary study endpoint of meeting the individualized treatment targets was also explored.

### Statistical analysis

Logistic regression and descriptive statistics were used to asses: (a) target reductions set by the investigators (overall and by country); (b) impact of baseline characteristics (age, frailty status, sex, screening HbA1c, duration of diabetes, and number of medications at baseline visit) on target setting (overall and by country); and (c) individualized HbA1c target response at endpoint (overall and by country). We also assessed the absolute change in HbA1c by country using a regression model with terms for treatment and centered baseline HbA1c. The odds ratio, defined as the odds of responding in one group divided by the odds of responding in the second group, was also presented. The last observation carried forward method was used to handle missing data because of early discontinuation or data censoring. Continuous data were used, wherever possible, to maximize power.

## Abbreviations

FAS: full analysis set
HbA1c: glycated hemoglobin
INTERVAL: INdividualized Treatment targets for EldeRly patients with type 2 diabetes using Vildagliptin Add-on or Lone therapy.
